# Cerebrospinal Fluid Cytokine and Chemokine Analysis in Dogs With Meningoencephalitis of Unknown Origin or Idiopathic Epilepsy

**DOI:** 10.1111/jvim.70229

**Published:** 2025-08-26

**Authors:** Michael J. Byron, Alison L. Parker, Stephen Parry, Yael Merbl

**Affiliations:** ^1^ College of Veterinary Medicine Cornell University Hospital for Animals Ithaca New York USA; ^2^ Cornell University Statistical Consulting Unit Ithaca New York USA

**Keywords:** cerebrospinal fluid, cytokine, dog, GM‐CSF, IFN‐γ, IL‐6, MCP‐1, MUE

## Abstract

**Background:**

Current diagnosis of brain disease in dogs is dependent on imaging and cerebrospinal fluid (CSF) analysis, including total nucleated cell counts and albumin concentrations.

**Hypothesis/Objectives:**

To determine whether multiplex cytokine/chemokine (Ct/Cm) analysis can differentiate among dogs with meningoencephalitis of unknown origin (MUE), idiopathic epilepsy (IE), and brain neoplasia.

**Animals:**

Client owned dogs diagnosed with brain disease with MRI and CSF diagnostics. Groups included 18 dogs with a diagnosis of MUE, 21 dogs with IE, and 7 dogs with brain tumors.

**Methods:**

A retrospective observational study; a multiplex immunoassay was utilized to measure CSF concentrations for the following: Interleukin (IL)‐2, IL‐6, IL‐7, IL‐8, IL‐10, IL‐15, and IL‐18, granulocyte macrophage colony‐stimulating factor (GM‐CSF), interferon gamma (IFN‐γ), keratinocyte chemoattractant (KC)‐like protein, IFN‐γ‐inducible protein‐10 (IP‐10), monocyte chemotactic protein 1 (MCP‐1), and tumor necrosis factor alpha (TNF‐α).

**Results:**

Several Ct/Cm were detected only in MUE cases: GM‐CSF (9/18), IFN‐γ (13/18), IL‐2 (8/18), IL‐15 (4/18), and TNF‐α (11/18). Other Ct/Cm concentrations were significantly higher in MUE cases (IL‐8: median 101 pg/mL, range 144, *p* = 0.019; IL‐18: median 3 pg/mL, range 0.52, *p* < 0.001; MCP‐1: median 814 pg/mL, range 1319, *p* = 0.004; and IL‐6: median 5 pg/mL, range 16, *p* < 0.001) compared to epilepsy and neoplasia.

**Conclusions and Clinical Importance:**

IFN‐γ, TNF‐α, GM‐CSF, IL‐2, and IL‐15 might be specific markers of MUE in canine CSF and could be potentially useful biomarkers in the diagnosis of MUE.

**Abbreviations**
CNS: central nervous system
CSF: cerebrospinal fluid
Ct/Cm: cytokine/chemokine
ELISA: enzyme‐linked immunosorbent assay
GM‐CSF: granulocyte macrophage colony‐stimulating factor
IE: idiopathic epilepsy
IFN‐γ: interferon gamma
IL: interleukin
IP‐10: IFN‐γ‐inducible protein‐10
KC‐like: keratinocyte chemoattractant‐like protein
MCP‐1: monocyte chemotactic protein 1
MRI: magnetic resonance imaging
MUE: meningoencephalitis of unknown origin
NME: necrotizing meningoencephalitis
TNCC: total nucleated cell count
TNF‐α: tumor necrosis factor alpha

## 1 Introduction

Seizures are one of the most common clinical signs of brain disorders in dogs, with a wide prevalence reported between different studies, ranging between 0.5% and 5% in non‐referral hospital populations [1, 2]. Common causes of seizures include idiopathic epilepsy (IE), brain neoplasia, and meningoencephalitis of unknown etiology (MUE) [3]. Identifying the primary cause of epileptic seizures can benefit the dog and maximize treatment options; however, many times it is not available.

Current clinical diagnosis of intracranial brain diseases is mainly dependent on magnetic resonance imaging (MRI) and cerebrospinal fluid (CSF) analysis. Specifically for IE, a diagnosis of exclusion is made based on lack of abnormal imaging and CSF cytologic findings [4]. However, overlap can exist between imaging findings ranging from a normal MRI appearance in MUE to ill‐defined hyperintensities in IE [5], which can pose difficulty in reaching a diagnosis. CSF analysis complements the MRI findings to increase diagnostic accuracy. The diagnostic value of MRI and current CSF variables in neurologic diseases in dogs is not high [6, 7].

Only 25% of MR images and 6% of CSF cytology yielded a diagnosis in cases that were not easily recognizable via imaging [6]. Another common limitation in clinical practice is financial constraint, which can prevent owners from pursuing cost‐prohibitive diagnostics. In such cases, a tentative diagnosis will be made based on the history, clinical signs, and the minimal database obtained. In some cases, this will include only a CSF analysis, due to either the financial constraints to conduct an MRI study or a brain biopsy, or a lack of availability to perform these diagnostic procedures.

Cytokines and chemokines (Ct/Cm) are protein messenger molecules involved in pro‐ and anti‐inflammatory states. Several cytokines have been explored individually in relation to MUE in dogs [8, 9, 10, 11], including IL‐12B, IL‐17, IL‐21, CCL19, and HMGB1. However, they have mainly been researched to extend our understanding of the pathogenic processes of MUE, and not compared to other etiologies to look for specificity for MUE [8]. In humans, a multiplex Ct/Cm assay has a good to excellent ability to serve as a potential diagnostic discriminator between inflammatory and non‐inflammatory CNS diseases, with 100% of the 43 cases tested assigned appropriately to their respective disease groups: infection, autoimmune, neoplasia, and controls, based on the Ct/Cm profiles [12].

The aim of this study was to use a bead‐based multiplex assay to determine whether the Ct/Cm profile in canine CSF can be used to distinguish MUE from IE or intracranial neoplasia.

## 2 Material and Methods

### 2.1 Case Selection

Medical records from dogs presenting to the Cornell University Hospital for Animals Neurology department were searched for those with a history of clinical signs indicating intracranial disease between 2023 and 2024. To meet the inclusion criteria, dogs were required to have had: (1) a complete neurological examination by a neurology resident and supervising neurology board specialist (either ACVIM or ECVN), (2) a brain MRI (1.5T MRI scanner, Vantage Orian, Canon Medical Systems, USA Inc. Tustin, CA, USA). Sequences obtained for clinical purposes included T1 weighted image (WI), T2WI, T2W Fluid‐attenuated inversion recovery (FLAIR), T2W gradient echo, T2W Short Tau Inversion Recovery (STIR) WI, and T1WI post contrast. Additionally, Diffusion‐weighted imaging (DWI) and a calculated Apparent Diffusion Coefficient (ADC) map were attained. A report by a board specialist radiologist (DACVR), a CSF analysis analyzed by a board specialist clinical pathologist (DACVP), and a clinical diagnosis of either MUE, IE/cryptogenic epilepsy (CE), or neoplasia were made based on the history, clinical signs, and the results of all diagnostic procedures. Dogs were tentatively diagnosed with MUE when an acute onset of clinical signs appeared. In most cases, multifocal signs were noted, and abnormal findings on an MRI study and/or CSF analysis were used to support the diagnosis. In a minority of cases—when both the CSF analysis and MRI were normal—an acute onset of clinical signs, progression of the disease, and signalment were the mainstay of clinical diagnosis. To be considered IE/CE, dogs had to have only seizures and had to be normal between events, with a lack of findings supporting other intracranial disease. Medical data collected included signalment, age of onset of clinical signs, time between onset of clinical signs and CSF tap, previous use of glucocorticoids, CSF analysis results, iatrogenic CSF blood contamination, and clinical outcomes.

To determine whether glucocorticoid use might affect CSF Ct/Cm concentrations, dogs were separated into two subgroups: (1) naïve to glucocorticoid since clinical sign onset; or (2) “not naïve,” i.e., receiving glucocorticoids at the time of tap. To determine whether time between “onset of clinical signs” and “time of tap” might influence Ct/Cm detection, dogs were also separated into two other subgroups: (1) ≤ 30 days between onset and tap; or (2) > 30 days between onset and tap.

### 2.2 Sample Collection

CSF was collected for each dog during the same anesthetic procedure conducted for MRI acquisition from either the occipito‐atlantal cistern or the lumbar region, as part of a standard clinical diagnostic workup. CSF samples were evaluated and analyzed by a board‐certified clinical pathologist to determine total nucleated cell count (TNCC), protein characteristics, and cell morphology. The remaining volume of the CSF sample was banked and frozen at −80°C and was available for multiplex Ct/Cm analyses. At the time of CSF collection, written consent was obtained from pet owners for use of leftover clinical samples for future research, which was approved by the Cornell Institutional Animal Care and Use Committee (IACUC protocol # 2022‐0134).

### 2.3 Cytokine Evaluation

All CSF samples were evaluated simultaneously for GM‐CSF, IFN‐γ, IL‐2, IL‐6, IL‐7, IL‐8, IL‐10, IL‐15, IL‐18, IP‐10, KC‐like, MCP‐1, and TNF‐α using a commercially available canine multiplex immunoassay (Milliplex MAP canine cytokine/chemokine assay, Millipore, Burlington, MA, USA) according to the manufacturer's instructions. This panel was chosen as a standard commercially available kit that has been used and validated for blood samples in dogs. This assay has also been previously reported to reliably measure cytokines and chemokines in canine CSF [13]. Briefly, antibody‐coated detection beads were incubated overnight at 4°C with supplied canine recombinant Ct and Cm standards, undiluted patient CSF samples, or quality controls in 96‐well plates. All wells were then incubated with biotinylated secondary detection antibodies followed by streptavidin‐phycoerythrin. After washing, wells were resuspended in 150 μL phosphate buffered saline and read immediately on a Luminex 200 instrument (Luminex, Austin, TX, USA). All standards, samples, and controls were run in duplicate. Mean fluorescence intensities were analyzed using Milliplex Analyst software (Millipore, Burlington, MA) to determine Ct/Cm concentrations for CSF samples from the within‐assay standards. All standards and controls were found to be within the expected concentration ranges.

### 2.4 Statistical Methods

Data were analyzed using R version ^1^ 4.4.0 (Puppy Cup). Ct/Cm concentrations were summarized as medians and IQR. Kruskal Wallis tests were used to compare median differences among groups, with Dunn's post hoc tests.^2^ Ct/Cm data were also categorized as detected or undetected, summarized into frequencies and percentages, and compared among diagnostic groups using Fisher's exact tests. Fisher's exact tests were also used to compare Ct or Cm detection rates among subgroups based on glucocorticoid use (Yes/No) and time from onset of signs to CSF collection (≤ or > 30 days). Additionally, logistic regression with disease as the outcome, CSF cytokines as the predictors, and time from onset to CSF tap as co‐variables were assessed. Statistical significance was established at *p* < 0.05 and was not adjusted for multiple comparisons in this study design.

## 3 Results

### 3.1 Study Groups: IE, MUE, and Neoplasia Clinical Data

Eighteen dogs with a clinical diagnosis of MUE were identified, with a median age of 6 years (range: 2–11 years old), of which six were neutered males, four intact males, and eight spayed females. Breeds included several mixed breeds (*n* = 6), Chihuahua (*n* = 4), French Bulldog (*n* = 2), Dachshund (*n* = 2), and one each of: Brussels Griffon, Pointer mix, Shih Tzu, and Boxer. The most common presenting clinical signs in dogs with MUE were seizures or seizure‐like activity (*n* = 6), signs of vestibular disease including head tilt, nystagmus, and nausea/vomiting (*n* = 6), ataxia or general proprioception deficits (*n* = 5), mentation changes (*n* = 3), and acute blindness (*n* = 2). The presenting clinical signs for each individual case are documented in Table S1.

There were 21 dogs with a clinical diagnosis of IE, with a median age of 6 years old (range 0.5–11 years old), of which 12 were neutered males, one intact male, and eight spayed females. Breeds included Labrador Retriever (*n* = 3), Goldendoodle (*n* = 2), Rottweiler (*n* = 2), Standard Poodle (*n* = 2), German Shepherd Dog (*n* = 2), and one each: Golden Retriever, Cairn Terrier, Doberman Pinscher, Cocker Spaniel, Border Collie, French Bulldog, Norwegian Elkhound, German Shorthaired Pointer, Great Pyrenees, and mixed breed dog. All dogs in the IE category had a history of seizures except for one that was suspected as either IE or fly biting syndrome not due to seizure (without an EEG to diagnose).

Seven dogs with a clinical diagnosis of intracranial neoplasia were identified, with a median age of 10 years (range 1–11 years old), of which six were neutered males and six spayed female. Breeds included one of each: Golden Retriever, Beagle, Boxer, French Bulldog, Great Dane, Labradoodle, and a mixed breed. Presentations included seizures (*n* = 5), facial nerve paresis (*n* = 1), and ataxia (*n* = 1). Individual clinical data are summarized in Table S1.

### 3.2 CSF Total Nucleated Cell Counts and Total Protein Concentrations

In dogs diagnosed with MUE, the results of total nucleated cell count (TNCC) and total protein (TP) varied considerably between dogs, ranging from markedly elevated counts to normal counts. All IE/CE dogs were found to have normal TNCC and TP concentrations. TNCC was within the normal reference range for all dogs with neoplasia, and TP was higher than normal in three of the cases, suggesting albumin‐cytologic dissociation.

### 3.3 CSF Cytokine and Chemokine Detection

All cytokines evaluated except for IP‐10 were detected in the CSF of at least one of the 46 dogs. In any given dog from any of the study groups, at least three cytokines were detected.

Five cytokines—IFN‐γ, TNF‐α, GM‐CSF, IL‐2, and IL‐15—were detected only in the CSF of MUE cases and not in IE or neoplasia cases (Table 1). Specifically, of the 18 dogs with MUE, IFN‐γ was detected in 13 dogs (72%). TNF‐α was detected in 11 (61%), GM‐CSF in 9 (50%), IL‐2 in 8 (44%), and IL‐15 in 4 (22%) MUE dogs. Of the 18 dogs with MUE, 14 dogs (78%) had detectable levels of at least one of these five cytokines.

**TABLE 1:** Proportion of CSF cytokines and chemokines detected using the multiplex assay in dogs with intracranial diseases.

Disease etiology (# of dogs)	Number of dogs with detected levels of Ct/Cm in their CSF												
	KC‐like	IL‐6	MCP‐1	IL‐7 (*p* = 0.005)	GM‐CSF (*p* < 0.001)	IFN‐γ (*P* < 0.001)	IL‐2 (*P* < 0.001)	IL‐15 (*p* = 0.027)	TNF‐a (*P* < 0.001)	IL‐8	IL‐10	IP‐10	IL‐18
MUE (18)	18	18	18	8	9	13	8	4	11	9	4	1	10
IE (21)	21	21	21	1	0	0	0	0	0	10	2	0	9
Neoplasia (7)	7	7	7	0	0	0	0	0	0	6	0	0	6

*Note:* Fisher's exact tests were used to assess the association between detection and group, with statistical significance set at *p* < 0.05.

**FIGURE 1:**
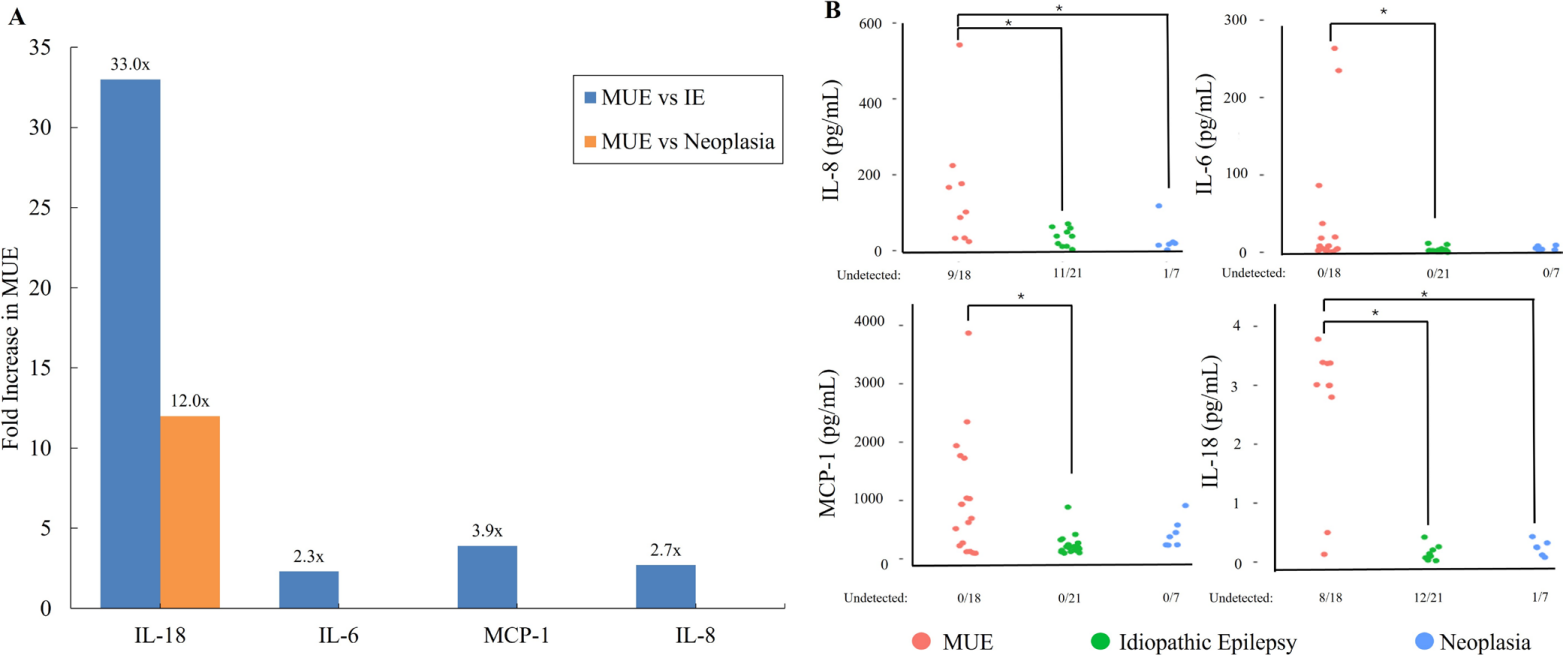
Cytokines and chemokines found to be significantly different between etiologies. Significance was determined by a Dunn's test after a significant Kruskal‐Wallis test. (A) Fold increase of median Ct/Cm concentrations between etiologies. (B) Scatter plots for IL‐8, IL‐6, MCP‐1, and IL‐18 show significant differences (*p* < 0.05) between the different etiologies. Numbers below each graph indicate the proportion of dogs that had Ct/Cm concentrations below the limit of detection for the assay.

Several cytokines were detected in more than one diagnostic group. IL‐6, MCP‐1, and KC‐like were detected in all 46 CSF samples evaluated. Concentrations of IL‐18 were around 30 times higher in MUE dogs (median 3 pg/mL, range 0.52 pg/mL) compared to IE (median 0.09, range 0.15 pg/mL) and 12 times higher compared to dogs with neoplasia (median 0.25 pg/mL, range 0.15 pg/mL); (*p* < 0.001; Table 2). Additionally, CSF IL‐6 was higher in dogs with MUE (median 4.71 pg/mL, range 15.7 pg/mL) compared to IE (median 2.05 pg/mL, range 1.05 pg/mL); (*p* = 0.007). MCP‐1 concentrations were three times higher in dogs with MUE (median 814.2 pg/mL, range 1319 pg/mL) compared to dogs with IE (median 207.46 pg/mL, range 98.2 pg/mL); (*p* = 0.008). IL‐8 concentrations were about three times higher in dogs with MUE (median 101 pg/mL, range 143.7 pg/mL) compared to dogs with IE (median 37 pg/mL, range 43.8 pg/mL; *p* = 0.019). Figure 1A depicts the comparative results between disease etiologies and the differences in cytokine concentration levels. Figure 1B shows the predictors scatter plots of the Ct/Cm based on disease etiology. Concentrations of the KC‐like cytokine did not differ between groups (*p* = 0.7). The size of the neoplasia group was small, limiting statistical power. Six dogs with MUE with normal CSF TNCC and TP had increased Ct/Cm concentrations.

**TABLE 2:** The concentrations of cytokines and chemokines (median and IQR) in pg/mL as measured in the different etiologies.

Cytokine	MUE, *N* = 18	Idiopathic epilepsy, *N* = 21	Neoplasia, *N* = 7	*p*
GM_CSF	1.34 (1.18)	0	0	
IFN‐γ	1 (4)	0	0	
IL‐2	1.65 (0.65)	0	0	
IL‐15	0.43 (0.77)	0	0	
TNF‐α	3 (1)	0	0	
IL‐8	101 (144)	37 (44)	18 (6)	0.019
IL‐18	3.00 (0.52)	0.09 (0.15)	0.25 (0.15)	< 0.001
MCP‐1	814 (1319)	207 (98)	378 (275)	0.004
IL‐6	5 (16)	2 (1)	4 (3)	< 0.001
IL‐10	9 (17)	2 (1)	NA (NA)	0.064
KC like	43 (105)	59 (42)	44 (48)	0.7
IL‐7	3.12 (1.28)	9.77 (0.00)	NA (NA)	0.12

*Note:* Kruskal–Wallis nonsignificant rank sum tests were used to compare median differences among groups. Values that were below the limit of detection were removed from the analysis. IP‐10 was excluded from further analysis because all samples but one were below the limit of detection. MUE, meningoencephalitis of unknown etiology. Light gray rows depict cytokines/chemokines only detected in MUE patients. Dark gray rows depict cytokines detected in all groups with significantly different concentrations, according to Dunn's test. White rows depict nonsignificant cytokine/chemokine concentrations among different groups.

### 3.4 Time of CSF Collection in Relation to the Onset of Clinical Signs

**FIGURE 2:**
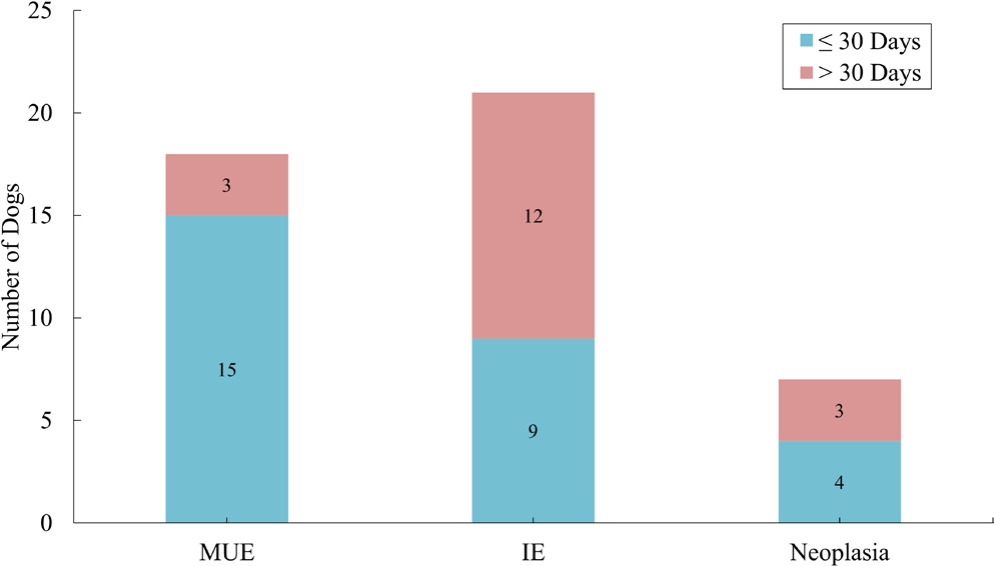
The timing of CSF collection (≤ 30 days vs. > 30 days) from the onset of clinical signs for each disease etiology.

Of the IE group, diagnosis and CSF collection of nine dogs occurred within the first 30 days of the onset of clinical signs, and for 12 dogs this occurred after 30 days (Figure 2). Fifteen of the MUE dogs were diagnosed within the first 30 days of onset of clinical signs, and three were diagnosed later. Of the neoplasia group, four dogs were diagnosed within the first 30 days and three were diagnosed later. Both IL‐6 and MCP‐1 concentrations were higher (2.43 times higher and 2.27 times higher, respectively) in dogs when the sample was conducted less than 1 month from the onset of clinical signs, compared to greater than 1 month. Additionally, IFN‐γ was detectable at a higher percentage when time to tap was less than 1 month. Only MCP‐1 showed a significant difference (*p* = 0.023) in time between diagnosis and collection of CSF in dogs with MUE.

### 3.5 Associations With Steroid Administration

One dog from the neoplasia group, no dogs from the IE group, and three dogs from the MUE group were categorized as not naïve to steroids at the time of CSF collection. Due to the low number of dogs treated with steroids, we were not able to draw any conclusions. Information regarding the specific dogs that were not naïve to steroids is available in Table S1.

### 3.6 CSF Iatrogenic Blood Contamination

For the Ct/Cm analytes that were always detectable (KC‐like, IL‐6, and MCP‐1), no significant difference was noted between samples with (*n* = 8) or without (*n* = 38) iatrogenic blood contamination (*p*‐values ranged from 0.056–1.000).

## 4 Discussion

In this study, we aimed to determine whether specific cytokines or chemokines in CSF could distinguish MUE from IE/CE or intracranial neoplasia in dogs. Of the 13 Ct/Cm tested, all but one had detectable levels in at least some dogs. Importantly, five cytokines (IFN‐γ, TNF‐α, GM‐CSF, IL‐2, and IL‐15) were only detected in MUE cases and could potentially be useful as MUE biomarkers. Additionally, only one chemokine (MCP‐1) was correlated with time from disease onset to collection of CSF.

IFN‐γ is involved in autoimmune brain diseases in humans, with evidence that it might play both pro‐inflammatory and disease‐limiting roles [8, 14]. In dogs, IFN‐γ is higher in the brain tissue of dogs with necrotizing meningoencephalitis (NME) [7]. IFN‐γ could act in a concentration‐dependent manner on microglia, with levels facilitating microglia neuroprotective functions, and high levels driving microglia toward inflammation [15].

TNF‐α, IL‐2, and IL‐15 were only found in the CSF of dogs with MUE in our study.

The time between the onset of clinical signs and CSF collection was significant, with an increase in both MCP‐1 and IL‐6 concentrations when the time to collection of CSF was under 1 month from the onset of clinical signs. However, when logistic regressions were performed for dogs with MUE, only MCP‐1 showed a significant difference. The source of MCP‐1 includes endothelium and peripheral macrophages [16]. CSF MCP‐1 concentrations are highest in the early phase of spinal cord injury, and it could have similar behavior in MUE, when in the initial phase there is a marked accumulation, and as the disease becomes more chronic, the concentrations decrease. Due to higher levels of MCP‐1 in the acute phase of the disease, the potential use of it as a biomarker to monitor relapse should be explored.

Detectability of changes in cytokine concentrations due to iatrogenic blood contamination or previous use of steroids at the time of collection of CSF was not found to be a significant confounding factor, although our sample sizes were small. The presence of blood contamination in CSF samples collected from infants minimally affects CSF cytokine values [17].

Cytokine concentrations differ between the CSF and systemic blood in brain diseases [18]. It is possible that cytokines and chemokines are produced mainly intrathecally in diseases that are confined to the CNS; thus, the peripheral blood contamination of the CSF does not tremendously affect the concentration of the cytokines involved in the CNS inflammatory process.

The use of steroids before CSF collection has previously been thought to alter results of CSF analysis, especially when evaluating for an inflammatory process, because of their anti‐inflammatory effects. Corticosteroids also decrease intracranial pressure [19] and decrease the permeability of the blood–brain barrier [20], which could alter CSF albumin concentrations. Our data did not show significant alteration in the Ct/Cm profiles of dogs that have been exposed to steroids compared to dogs that have not. However, only a few cases were not naïve to steroids, so it was not possible to draw any clinical conclusions.

A recent study in dogs reported a sensitivity and specificity of almost 100% for differentiating between MUE and intracranial neoplasia, based on individual binding patterns of serum antibodies and thousands of random peptides in the serum [21]. This study's findings suggest that different disorders might have unique immune signatures, thus easily delineating the diseases involved. However, due to cost and time factors, this accurate immune‐signature technique is not feasible in a clinical veterinary setting. Further investigations are needed to determine whether Ct/Cm profiles measured after CSF sampling can yield similar levels of sensitivity and specificity, while also allowing for more clinical feasibility.

This study has several limitations, which include a small sample size, especially in the neoplasia group. We cannot make solid conclusions regarding using CSF Ct/Cm to differentiate MUE and neoplasia, and future studies will need to assess the differentiating potential of CSF cytokines for different disease etiologies including but not limited to neoplasia. A second limitation lies in the lack of definitive diagnoses such as a biopsy or a histopathology report. Our facility does not conduct brain biopsies, and therefore we are dependent on a clinical diagnosis based on the diagnostic procedures at hand, such as a CSF analysis and an MRI. A recent study assessed the diagnostic accuracy of MUE cases based on a clinical diagnosis compared to histopathology reports and reported that up to 20.4% of the suspected MUE cases received a diagnosis of neoplasia or vascular insults [22].

We tried to minimize the timeframe of sample collections in this study to avoid potential degradation of the cytokines. This results in a lack of longer follow‐up times that would potentially differentiate between the MUE and neoplasia patients who are less likely to survive long term.

Lastly, our study includes CSF sampling from both the cisternal and lumbar sites. Since human CSF is sampled from the lumbar region only, we have not found literature evidence that both sampling sites will have similar concentrations. It is unknown whether sampling from both sites has confounded the results.

Because of the retrospective nature of the study, all CSF samples were frozen and then thawed before analysis, so concentrations detected in fresh CSF samples might differ. Ct/Cm stability in CSF has not been extensively addressed, and it is suspected that the freeze–thaw cycles affect the protein component of CSF [23]. However, our samples were collected and saved for less than 3 years and only thawed once for the purpose of this study.

## Disclosure

Authors declare no off‐label use of antimicrobials.

## Ethics Statement

Approved by the Institutional Animal Care and Use Committees (IACUC) at Cornell University College of Veterinary Medicine (IACUC protocol # 2022‐0134). Authors declare human ethics approval was not needed.

## Conflicts of Interest

The authors declare no conflicts of interest.

## Supporting information


**Table S1:** Clinical signs for each individual case.

## Endnotes

^1^
R Core Team (2024). R: A Language and Environment for Statistical Computing_. R Foundation for Statistical Computing, Vienna, Austria. <https://www.R‐project.org/>.

^2^
Dinno A 2024 Dunn.test: Dunn's Test of Multiple Comparisons Using Rank Sums_. R package version 1.3.6, https://CRAN.R‐project.org/package=dunn.test.
